# Supplementary figures and images for: The injured sciatic nerve atlas (iSNAT), insights into the cellular and molecular basis of neural tissue degeneration and regeneration
Source: eLife. 2022 Dec 14;11:e80881. doi: 10.7554/eLife.80881 (PMC9829412; doi:10.7554/eLife.80881)

WT Naive  
10/22/21

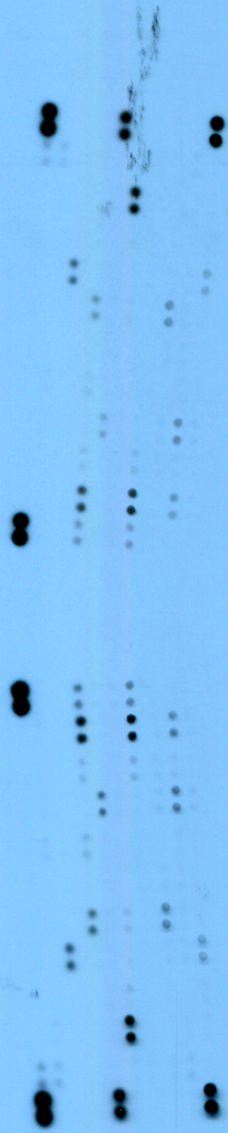

30 sec

1 min

2 min

5 min

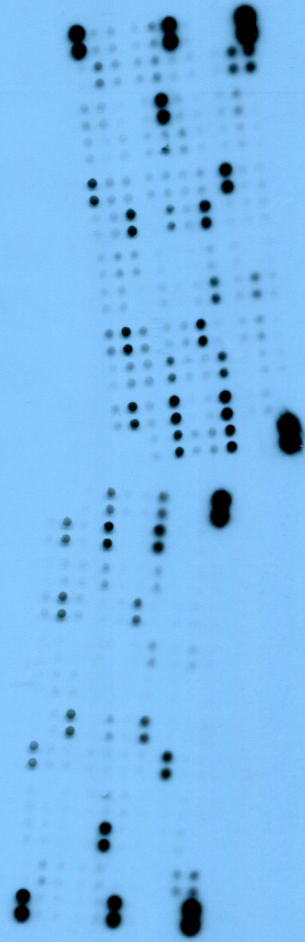

Supplement: Figure 1—figure supplement 1—source data 1. [file elife-80881-fig1-figsupp1-data1.zip › Fig-1_Suppl-3-source data_ELISA original X-ray films/Fig1-Supp3-A_source data_ELISA of WT-Naive-Sciatic-Nerve.pdf]

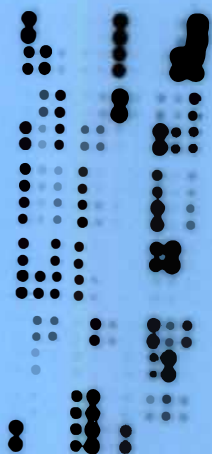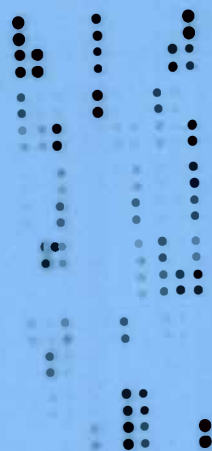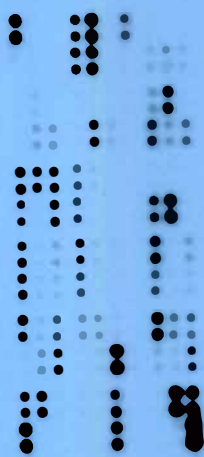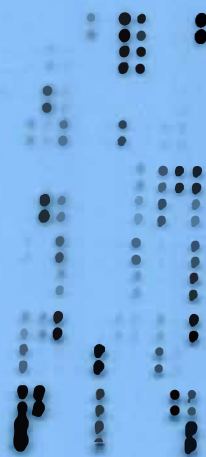

Supplement: Figure 1—figure supplement 1—source data 1. [file elife-80881-fig1-figsupp1-data1.zip › Fig-1_Suppl-3-source data_ELISA original X-ray films/Fig1-Supp3-D_source data_ELISA of WT-Naive-Blood-Serum.pdf]

WT  
 SNC  
 1opi  
 Injury  
 Site  
 1/12/22  
 200mg

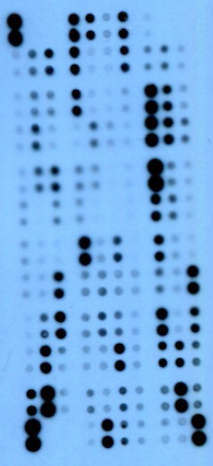

2 minute

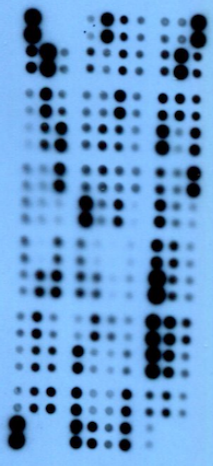

5 minute

~~200mg~~

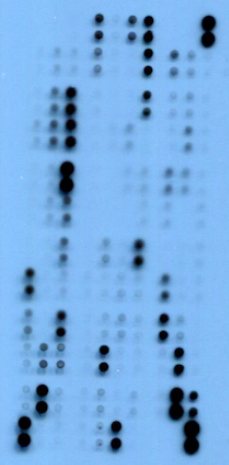

1 minute

~~200mg~~

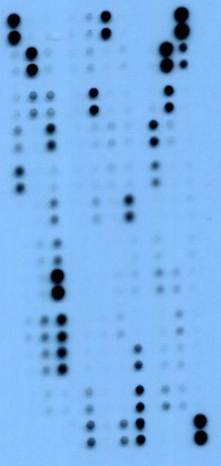

30 seconds

Supplement: Figure 4—figure supplement 1—source data 1. [file elife-80881-fig4-figsupp1-data1.zip › Fig-4_Suppl-1-source data_ELISA original X-ray films/Fig4-Supp1-A_source data_ELISA of 1dpc-Sciatic-Nerve.pdf]

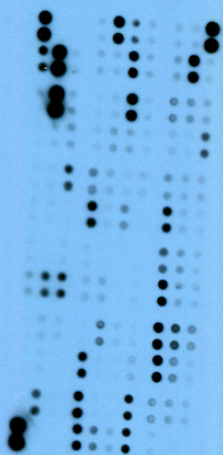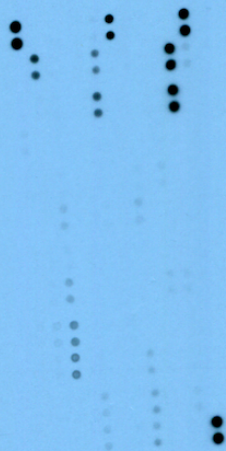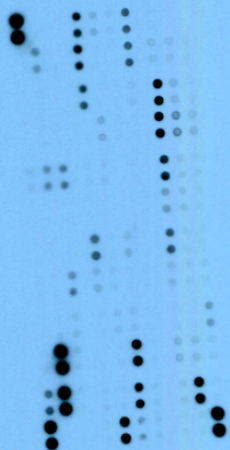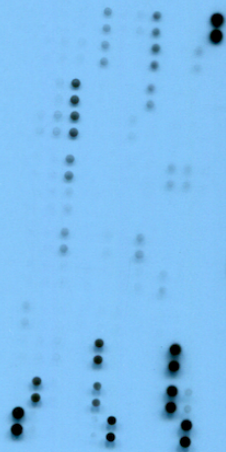

Supplement: Figure 4—figure supplement 1—source data 1. [file elife-80881-fig4-figsupp1-data1.zip › Fig-4_Suppl-1-source data_ELISA original X-ray films/Fig4-Supp1-B_source data_ELISA of 3dpc-Sciatic-Nerve.pdf]

1 2 3 4 5 6 7 8 9 10 11 12 13 14 15 16 17 18 19 20 21 22 23 24 25 26 27 28 29 30 31 32 33 34 35 36 37 38 39 40 41 42 43 44 45 46 47 48 49 50 51 52 53 54 55 56 57 58 59 60 61 62 63 64 65 66 67 68 69 70 71 72 73 74 75 76 77 78 79 80 81 82 83 84 85 86 87 88 89 90 91 92 93 94 95 96 97 98 99 100 101 102 103 104 105 106 107 108 109 110 111 112 113 114 115 116 117 118 119 120 121 122 123 124 125 126 127 128 129 130 131 132 133 134 135 136 137 138 139 140 141 142 143 144 145 146 147 148 149 150 151 152 153 154 155 156 157 158 159 160 161 162 163 164 165 166 167 168 169 170 171 172 173 174 175 176 177 178 179 180 181 182 183 184 185 186 187 188 189 190 191 192 193 194 195 196 197 198 199 200 201 202 203 204 205 206 207 208 209 210 211 212 213 214 215 216 217 218 219 220 221 222 223 224 225 226 227 228 229 230 231 232 233 234 235 236 237 238 239 240 241 242 243 244 245 246 247 248 249 250 251 252 253 254 255 256 257 258 259 260 261 262 263 264 265 266 267 268 269 270 271 272 273 274 275 276 277 278 279 280 281 282 283 284 285 286 287 288 289 290 291 292 293 294 295 296 297 298 299 300 301 302 303 304 305 306 307 308 309 310 311 312 313 314 315 316 317 318 319 320 321 322 323 324 325 326 327 328 329 330 331 332 333 334 335 336 337 338 339 340 341 342 343 344 345 346 347 348 349 350 351 352 353 354 355 356 357 358 359 360 361 362 363 364 365 366 367 368 369 370 371 372 373 374 375 376 377 378 379 380 381 382 383 384 385 386 387 388 389 390 391 392 393 394 395 396 397 398 399 400 401 402 403 404 405 406 407 408 409 410 411 412 413 414 415 416 417 418 419 420 421 422 423 424 425 426 427 428 429 430 431 432 433 434 435 436 437 438 439 440 441 442 443 444 445 446 447 448 449 450 451 452 453 454 455 456 457 458 459 460 461 462 463 464 465 466 467 468 469 470 471 472 473 474 475 476 477 478 479 480 481 482 483 484 485 486 487 488 489 490 491 492 493 494 495 496 497 498 499 500 501 502 503 504 505 506 507 508 509 510 511 512 513 514 515 516 517 518 519 520 521 522 523 524 525 526 527 528 529 530 531 532 533 534 535 536 537 538 539 540 541 542 543 544 545 546 547 548 549 550 551 552 553 554 555 556 557 558 559 560 561 562 563 564 565 566 567 568 569 570 571 572 573 574 575 576 577 578 579 580 581 582 583 584 585 586 587 588 589 590 591 592 593 594 595 596 597 598 599 600 601 602 603 604 605 606 607 608 609 610 611 612 613 614 615 616 617 618 619 620 621 622 623 624 625 626 627 628 629 630 631 632 633 634 635 636 637 638 639 640 641 642 643 644 645 646 647 648 649 650 651 652 653 654 655 656 657 658 659 660 661 662 663 664 665 666 667 668 669 670 671 672 673 674 675 676 677 678 679 680 681 682 683 684 685 686 687 688 689 690 691 692 693 694 695 696 697 698 699 700 701 702 703 704 705 706 707 708 709 710 711 712 713 714 715 716 717 718 719 720 721 722 723 724 725 726 727 728 729 730 731 732 733 734 735 736 737 738 739 740 741 742 743 744 745 746 747 748 749 750 751 752 753 754 755 756 757 758 759 760 761 762 763 764 765 766 767 768 769 770 771 772 773 774 775 776 777 778 779 780 781 782 783 784 785 786 787 788 789 790 791 792 793 794 795 796 797 798 799 800 801 802 803 804 805 806 807 808 809 810 811 812 813 814 815 816 817 818 819 820 821 822 823 824 825 826 827 828 829 830 831 832 833 834 835 836 837 838 839 840 841 842 843 844 845 846 847 848 849 850 851 852 853 854 855 856 857 858 859 860 861 862 863 864 865 866 867 868 869 870 871 872 873 874 875 876 877 878 879 880 881 882 883 884 885 886 887 888 889 890 891 892 893 894 895 896 897 898 899 900 901 902 903 904 905 906 907 908 909 910 911 912 913 914 915 916 917 918 919 920 921 922 923 924 925 926 927 928 929 930 931 932 933 934 935 936 937 938 939 940 941 942 943 944 945 946 947 948 949 950 951 952 953 954 955 956 957 958 959 960 961 962 963 964 965 966 967 968 969 970 971 972 973 974 975 976 977 978 979 980 981 982 983 984 985 986 987 988 989 990 991 992 993 994 995 996 997 998 999 1000 1001 1002 1003 1004 1005 1006 1007 1008 1009 1010 1011 1012 1013 1014 1015 1016 1017 1018 1019 1020 1021 1022 1023 1024 1025 1026 1027 1028 1029 1030 1031 1032 1033 1034 1035 1036 1037 1038 1039 1040 1

**Figure 6**

Supplement: Figure 9—source data 1. [file elife-80881-fig9-data1.zip › Fig-9-source data-original western blot associted with Fig-9P/Fig-9_source data_CD11b detected by Western Blot for sciatic nerve samples as shown in Fig9P.pdf]

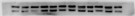

Supplement: Figure 9—source data 1. [file elife-80881-fig9-data1.zip › Fig-9-source data-original western blot associted with Fig-9P/Fig-9_source data_ERK detected by Western Blot for sciatic nerve samples as shown in Fig9P.pdf]
