# Supplementary material for: The injured sciatic nerve atlas (iSNAT), insights into the cellular and molecular basis of neural tissue degeneration and regeneration
Source: eLife. 2022 Dec 14;11:e80881. doi: 10.7554/eLife.80881 (PMC9829412; doi:10.7554/eLife.80881)
Supplement: Figure 4—figure supplement 1—source data 1. [file elife-80881-fig4-figsupp1-data1.zip › Fig-4_Suppl-1-source data_ELISA original X-ray films/Fig4-Supp1-C_source data_ELISA of 7dpc-Sciatic-Nerve.pdf]

Injury
