# Supplementary material for: The injured sciatic nerve atlas (iSNAT), insights into the cellular and molecular basis of neural tissue degeneration and regeneration
Source: eLife. 2022 Dec 14;11:e80881. doi: 10.7554/eLife.80881 (PMC9829412; doi:10.7554/eLife.80881)
Supplement: Supplementary file 1. — The table shows newly generated and existing scRNAseq datasets used in this study. Columns show cell numbers before and after applying exclusion criteria, replicates, statistics on reading depth and sequence saturation. A total of 157,409 high-quality single-cell transcriptomes were analyzed from naïve mouse sciatic nerve, injured sciatic nerves, and peripheral blood mononuclear cells (PBMC). Some of the 3 day (3d) injured nerves were divided into injury site and distal nerve and sequenced separately. SN, sciatic nerve; UMI, unique molecular identifier. [file elife-80881-supp1.docx]

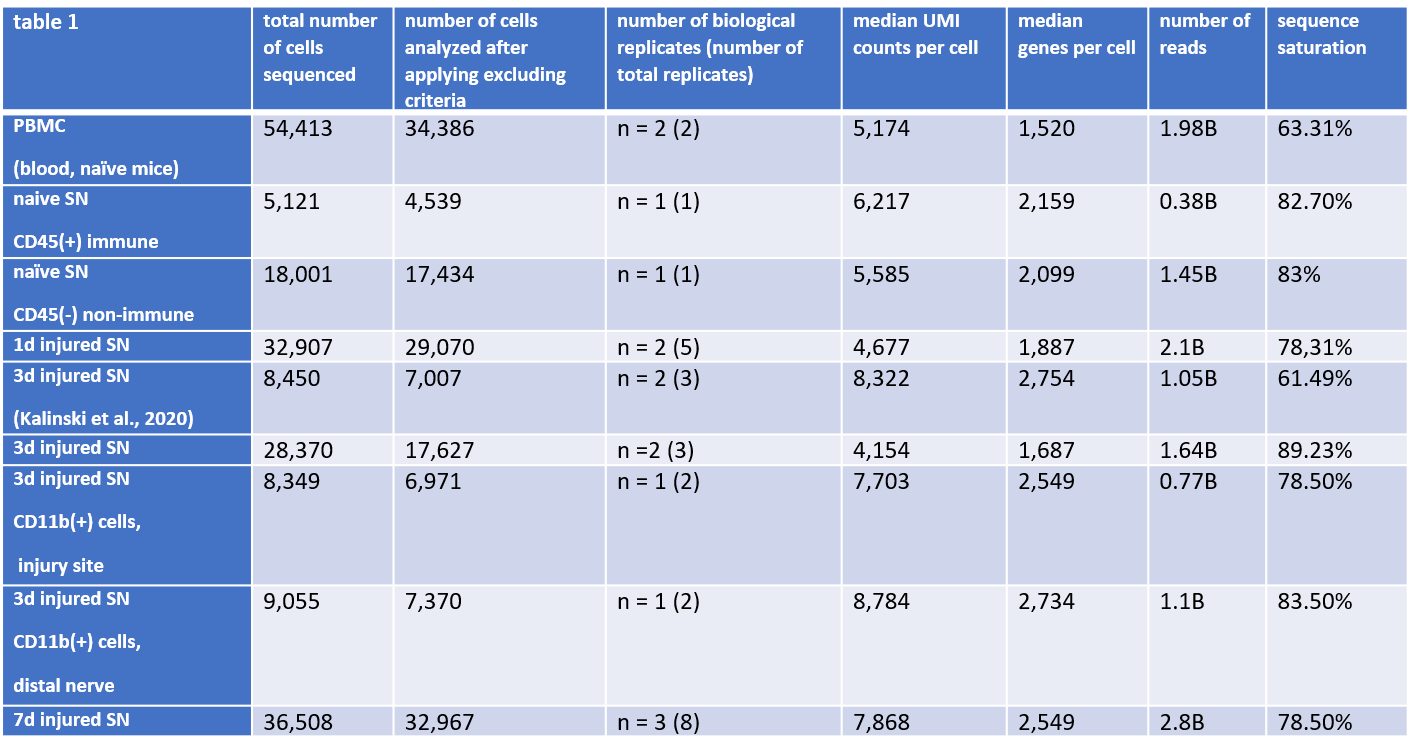


**Table. S1**

Datasets included in iSNAT. The table shows newly generated and existing scRNAseq datasets used in this study; cell numbers, replicates, statistics on reading depth and sequence saturation. A total of 157,371 high-quality single cell transcriptomes were analyzed from naïve mouse sciatic nerve, injured sciatic nerve, and PBMC. Some of the 3-day (3d) injured nerves were divided into injury site and distal nerve and sequenced separately. Abbreviations, SN (sciatic nerve), PBMC (peripheral blood mononuclear cells), UMI (unique molecular identifier).
